# Rapid increase in SARS-CoV-2 seroprevalence during the emergence of Omicron variant, Finland

**DOI:** 10.1007/s10096-022-04448-x

**Published:** 2022-04-28

**Authors:** Maarit J. Ahava, Hanna Jarva, Anne J. Jääskeläinen, Maija Lappalainen, Olli Vapalahti, Satu Kurkela

**Affiliations:** 1 HUS Diagnostic Center, HUSLAB, Clinical Microbiology, University of Helsinki and Helsinki University Hospital, P.O.B. 720 (Topeliuksenkatu 32), N00029 HUS Helsinki, Finland; 2 Translational Immunology Research Program and Department of Bacteriology and Immunology, University of Helsinki, Helsinki, Finland; 3 Department of Virology, Faculty of Medicine, University of Helsinki, Helsinki, Finland; 4 Department of Veterinary Biosciences, Faculty of Veterinary Medicine, University of Helsinki, Helsinki, Finland

The emergence of SARS-CoV-2 Omicron variant (B.1.1.529) as of November 2021 changed the epidemiology of COVID-19 with rapid upsurge of cases globally [1, 2]. In Finland, the first patient case with Omicron variant was detected on 29 November 2021 [3]. In this study, we conducted a prospective seroepidemiological survey of SARS-CoV-2 in November 2021–March 2022 in the Greater Helsinki area, Finland. Our aim was to assess changes in exposure and prevalence of SARS-CoV-2 infection during the first months of emergence of Omicron variant.

The study was institutionally approved (HUS/56/2021). Altogether 1,600 serum specimens were analyzed with Abbott SARS-CoV-2 IgG II Quant (IgG antibodies to receptor-binding domain (RBD) of the S1 subunit of the S protein of SARS-CoV-2) and N antibodies with Abbott SARS-CoV-2 IgG (IgG antibodies to N protein of SARS-CoV-2) on the Alinity i analyzer [4, 5]. The sampling scheme included 100 specimens each week between weeks 46/2021 and 9/2022, from routine samples sent to HUS Diagnostic Center, Helsinki. The sampling frame consisted of the ca 17,000 serum specimens that were sent for routine diagnostic purposes to HUS Diagnostic Center for HIV screening between 15 November 2021 and 6 March 2022 and were negative for HIV Ag/Ab. Samples were stored according to date of specimen. To select samples for each calendar week, a random starting point was chosen, and specimens were systematically selected until 100 specimens plus 5 spare samples were reached: the chosen 100 specimens were analyzed for SARS-CoV-2 antibodies. If the analysis failed or the specimen volume was not adequate, the sample was replaced by one of the spare samples of that calendar week.

The subgroups identified according to serostatus were (I) anti-N negative, anti-S1 negative: no serological evidence of vaccine immunization or previous infection; (II) anti-N negative, anti-S1 positive: seroresponse to vaccine immunization, no evidence of recent infection; (III) anti-N positive, anti-S1 positive: consistent with previous infection, vaccine immunization status unknown; (IV) anti-N positive, anti-S1 negative: recent infection possible, no evidence of vaccine immunization. The proportion of these subgroups was determined for each calendar week and statistical analyses were performed using IBM SPSS statistical program package, version 25. Visualization was done with GraphPad Prism 8.0.1.

The study subjects’ age ranged from 11 months to 94 years (median 33 years; IQR 26–46 years), and the proportion of women was 55.2%. The baseline prevalence of N antibodies in the first five weeks of the study period (46–50/2021) was 5.2%, while in the final 5 weeks (5–9/2022) it was 28.2%. The proportion of seronegative samples for the corresponding time frames was 11.6% and 3.8%, and for anti-N-negative, anti-S1-positive samples 84.2% and 68.2%. Figure 1 depicts the moving average of the N antibody seroprevalence over the study period: the sharpest increase was observed in those aged < 30 years. In late 2021, the seroprevalence of N antibodies was consistently well below 10% but began a rapid incline as of week 1/2022 and surpassed 20% on week 3/2022.

**Fig. 1:**
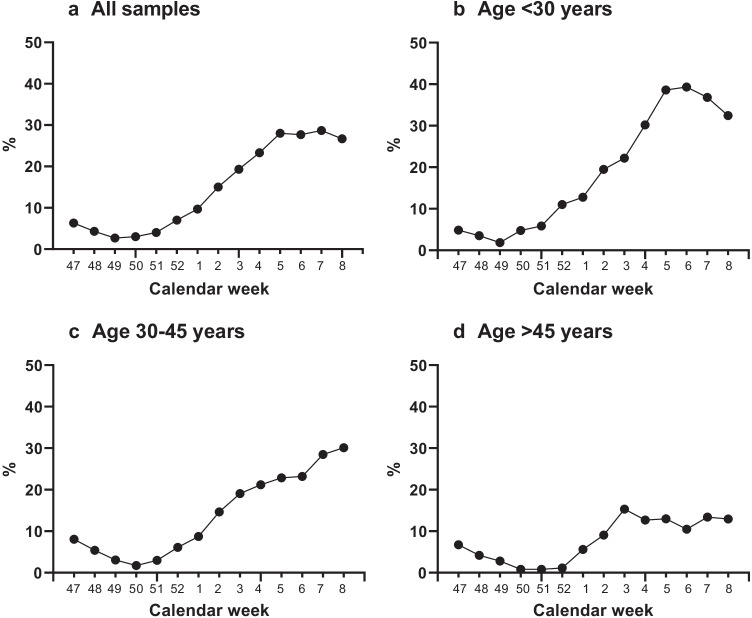
Proportion of N antibody–positive samples, 3-week moving average. **a** All samples from all age groups, *N* = 1600. **b** Study subjects under 30 years, *N* = 616. **c** Study subjects 30–45 years, *N* = 580. **d** Study subjects over 45 years, *N* = 404

Anti-N-positive samples that were anti-S1 negative began to appear on week 2/2022 and represented 0.9% (14/1600) of all analyzed samples, which may reflect a diminished or delayed seroresponse against S1 during Omicron infection. The increase in anti-N-positive samples (groups III and IV) was reflected as a decreasing proportion of seronegative samples (group I) towards the end of the study period. The proportions of subgroups (I–IV) per calendar week are presented in Fig. 2.

**Fig. 2:**
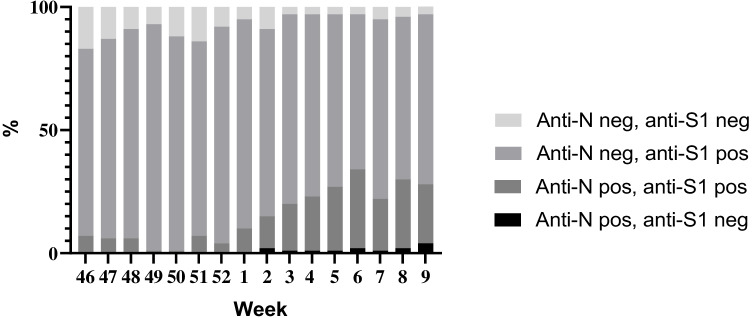
Proportions of subgroups over the study period. The increase in N antibody–positive samples was reflected as a decreased proportion of both the group of completely seronegative samples and samples of immunized individuals

By mid-December 2021, Omicron had become the primary variant in the Greater Helsinki area [3]. Soon after, our data show a rapid increase in the population level exposure to SARS-CoV-2. This indicates a high transmission rate and is in line with previous reports from elsewhere [6–8 ]. At the end of the study period (week 9/2022), 4% were seronegative in N and S antibody testing. Altogether, 78% (1241/1600) had S antibodies without N antibodies, suggesting vaccine immunization without recent COVID-19 infection. In the Greater Helsinki area, 79% had received at least one SARS-CoV-2 vaccine at the time of writing, including all age groups [9].

Our study design did not allow differentiation between those who had undergone COVID-19 with or without prior vaccine immunization. Also, protective immunity against SARS-CoV-2 cannot be determined by detection of antibodies to N or S antigen by enzyme immunoassays. As the purpose of this study was to provide real-time data, conducting neutralization assays was not feasible.

The present study showed a rapid increase in the N antibody prevalence, indicating that approximately 23% (comparing the first and last 5 weeks of the study period) of the tested individuals had contracted SARS-CoV-2 infection during the emergence of Omicron variant. While our sampling frame does not perfectly reflect the general population, the data suggest that during the study period, well beyond 300,000 individuals in the Greater Helsinki area (population 1.5 million inhabitants) underwent COVID-19, while at the same time approximately 230,000 COVID-19 cases were officially diagnosed in Greater Helsinki [9]. The present study suggests that a substantial proportion of COVID-19 cases remained undiagnosed during the emergence of Omicron, probably due to subclinical infections and diminished RT-PCR testing.

## Data Availability

The datasets generated during and/or analyzed during the current study are available from the corresponding author.
